# Monoterpenoid indole alkaloids from *Alstonia rostrata*

**DOI:** 10.1007/s13659-012-0019-y

**Published:** 2012-04-19

**Authors:** Mei-Fen Bao, Chun-Xia Zeng, Yan Qu, Ling-Mei Kong, Ya-Ping Liu, Xiang-Hai Cai, Xiao-Dong Luo

**Affiliations:** 1 State Key Laboratory of Phytochemistry and Plant Resources in West China, Kunming Institute of Botany, Chinese Academy of Sciences, Kunming, 650201 China; 2 Southwest China Germplasm Bank of Wild Species, Kunming Institute of Botany, Chinese Academy of Sciences, Kunming, 650201 China; 3 Graduate University of Chinese Academy of Sciences, Beijing, 100049 China

**Keywords:** monoterpenoid indole alkaloid, alstrostines C-F, *Alstonia rostrata*, Apocynaceae

## Abstract

Four new monoterpenoid indole alkaloids, alstrostines C-F together with thirteen known alkaloids were isolated from the leaves and twigs of *Alstonia rostrata*. All structures of new compounds were elucidated based on NMR, FTIR, UV, and MS spectroscopic data. Alstrostines C-E might originate from keto-enol tautomerism of preakummicine during biogenetic pathway of akummicine.

**Electronic Supplementary Material:**

Supplementary material is available for this article at 10.1007/s13659-012-0019-y and is accessible for authorized users.

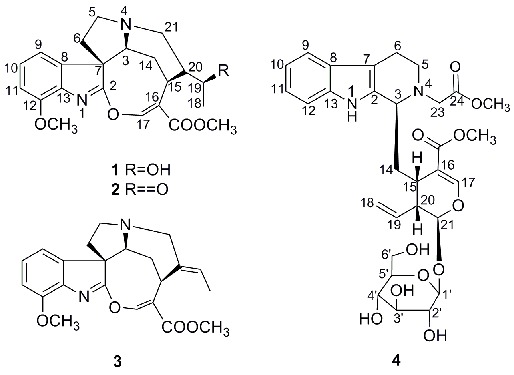

## Introduction

Monoterpenoid indole alkaloids (MIAs), including more than 2000 compounds, are classified to five types, corynanthe, strychnos, iboga, aspidosperma, and yohimbinoid, which play a very important role in natural medicinal history.[1] The genus *Alstonia* of Apocynaceae is rich of monoterpenoid indole alkaloids, and eight species of the genus are distributed in Yunnan province.[2] The phytochemical constituents of *Alstonia* sp. have been investigated intensively with anticancer, antibacterial, antifertility, and antitussive activity being reported.[3] We reported new alkaloids from *A. scholaris*, and *A. yunnanensis*, and *A. mairei* recently.[4] As part of systematic phytochemical research on Yunnan endemic resources, another species, *A. rostrata*, named *Winchia calophylla* in *Flora of China*,[5] was also investigated. Previous studies on the stem bark and root of *A. rostrata*, collected from Yunnan Province of China, have reported a number of indole alkaloids and some nonalkaloid compounds.[6] In the current study, separation of total alkaloids led to seventeen MIAs besides alstrostines A and B.[7] In this paper, we will describe the isolation and structural elucidation of other four new alkaloids alstrostines C–F (**1**–**4**) together with thirteen known isolates, 19,20-dihydroakuammicine (**5**), echitamidine (**6**), 12-methoxyechitamidine (**7**), 19-oxo-12-methoxyechitamidine, vallesiachotamine (**9**), isovallesiachotamine (**10**), deacetylakuammiline (**11**), 17-*O*-acetyl-$N^4$-demethylechitamine (**12**), $N^4$-demethylechitamine (**13**), akuammidine (**14**), 6,7-secoangustilobine (**15**), undulifoline (**16**), tabersonine (**17**). The biogenetic pathway of the new alkaloids was proposed. In addition, all compounds were tested for their cytotoxicity against five human cancer cell lines, but no activity was found ($IC_{50}$ > 40 $\mu$M).

**Figure 1.** New alkaloids from *Alstonia rostrata*

*To whom correspondence should be addressed. E-mail: xhcai@mail.kib.ac.cn (X.H. Cai); xdluo@mail.kib.ac.cn (X.D. Luo).

## Results and Discussion

The MeOH extract of *A. rostrata* leaves and twigs was partitioned between $H_2O$ and EtOAc after acid-alkali treating, and column chromatography was used to separate the alkaloidal fraction into seventeen alkaloids.

Alkaloid **1** gave positive reactions with Dragendorff' reagent and had a molecular formula of $C_{22}H_{26}N_2O_5$ by HRESIMS at *m/z* 399.1919 [M + H]$^+$. The maximum UV absorptions at 214, 275, and 326 nm of **1** pointed to an indole alkaloid with a *β*-anilineacrylate system[8] in agreement with the FTIR absorption bands at 3453, 1705, and 1646 cm$^{-1}$. In the $^1$H NMR spectrum of **1,** three signals [$\delta_H$ 6.98 (d, *J* = 8.0 Hz, H-9), 7.13 (t, *J* = 8.0 Hz, H-10), 7.03 (d, *J* = 8.0 Hz, H-11) revealed the presence of a mono-subsitituted A ring in MIA.[6b] The $^{13}$C NMR and DEPT spectra of **1** indicated signals for a dihydroindole ring [$\delta_C$ 128.5 (s, C-13), 139.7 (s, C-8), 112.3 (d, C-11), 113.4 (d, C-9), 126.7 (d, C-10), 148.3 (s, C-12), 55.0 (s, C-7)]. Moreover, **1** also possessed three methylenes [$\delta_C$ 53.8 (C-5), 41.6 (C-6), 48.5 (C-21)], three methines [$\delta_C$ 68.5 (C-19), 59.9 (C-3), 31.5 (C-15)], one methyl ($\delta_C$ 20.9, C-18), and one sp$^2$ quaternary carbon ($\delta_C$ 152.0) besides confirming presence of methyl *β*-anilineacrylate conjugation [$\delta_C$ 170.5 (s), 160.0 (d), 53.8 (q), 120.1 (s)][8]. The quaternary carbon signal at $\delta_C$ 152.0 correlated with H-3 ($\delta_H$ 3.75) and H-6 ($\delta_H$ 1.65 and 2.82) in the HMBC spectrum was assigned to C-2. Detailed analysis of $^{13}$C NMR and DEPT data revealed **1** might be belong to akuammicine-type alkaloids. Further NMR comparison with those of 12-methoxyechitamidine (**7**)[9] indicated that **1** was similar to **7** with exception for an additional methine ($\delta_C$ 160.0 and $\delta_H$ 9.12) in **1**. The methine proton showed HMBC correlation to C-2, suggesting that the methine was connected with C-2 by an oxygen bridge with consideration of its molecular formula.

The UV spectrum of **2** and **3** also indicated the absorption bands of indole rings with a *β*-anilineacrylate system together with FTIR spectrum. Compound **2** was found to possess a molecular formula of $C_{22}H_{24}N_2O_5$ as evidenced by HRESIMS at *m/z* 397.1763 [M + H]$^+$, an additional degree of unsaturation to **1**. The $^1$H NMR spectra of **2** also displayed the signals for mono-substituted indole ring [$\delta_H$ 7.00 (d, *J* = 7.9 Hz, H-9), 7.05 (d, *J* = 7.9 Hz, H-11), 7.16 (t, *J* = 7.9 Hz, H-10). Its $^{13}$C NMR and DEPT data showed similar pattern to **1** (see Table **1**) except that the methine of C-19 was disappeared, instead a new signal of carbonyl group ($\delta_C$ 207.2, s) was present in **2**. Its HMBC correlations could support it, in which $\delta_H$ 2.24 (H-18) was correlated with $\delta_C$ 207.2 (C-19) and 50.6 (C-18). Compound **3** possessed a molecular formula of $C_{22}H_{24}N_2O_4$ based on the HRESIMS. The $^1$H and $^{13}$C NMR spectra of **3** displayed similarity to **1** except for two methine signals at $\delta_C$ 68.5 (C-19) and 47.3 (C-20) in **1** were substituted by double bond signals ($\delta_C$ 136.1 and 119.8) in **3**, suggesting that **3** was a dehydration product of **1**.

Configuration of alkaloids **1**–**3** was determined by NMR and ROESY spectra together with their biogenetic pathway. The chemical shift of C-19 in **1** was deshielded (Δ2.6 ppm) similar to $N^4$-demethyl-12-methoxyalstogustine and $N^4$-demethylalstogustine, relative to 12-methoxyechitamidine and echitamidine due to intramolecular H-bonding between the nitrogen atom and the C-19-OH.[6a] The ROESY correlations of H-3 with H-15 and H-20 in **1**–**3** placed them on the same sides. The double bond C-19/20 of **3** was determined as *E* according the ROESY correlation of H-19 with H-21. In the biogenetic pathway of akummicine,[10] the keto-enol tautomerism among O-$C_{17}$-$C_{16}$ of preakuammicine would give two reaction routes, which led to alstrostines C–E and akummicine, respectively

**Table 1.** $^{13}$**C NMR spectroscopic data for compounds 1–4 (1–3 in acetone-*d*$_6$, 4 in methanol-*d*$_4$, *δ* in ppm, *J* in Hz)**

| carbon | 1 | 2 | 3 | 4 |
|---|---|---|---|---|
| 2 | 152.0 s | 152.0 s | 152.0 s | 136.1 s |
| 3 | 59.9 d | 59.9 d | 60.5 d | 58.6 d |
| 5 | 53.8 t | 53.8 t | 54.1 t | 44.5 t |
| 6 | 41.6 t | 41.5 t | 42.1 t | 17.5 t |
| 7 | 55.0 s | 55.1 s | 55.3 s | 107.5 s |
| 8 | 139.7 s | 139.5 s | 139.8 s | 128.2 s |
| 9 | 113.4 d | 113.3 d | 113.6 d | 118.5 d |
| 10 | 126.7 d | 126.8 d | 126.6 d | 119.4 d |
| 11 | 112.3 d | 112.4 d | 112.3 d | 121.7 d |
| 12 | 148.3 s | 148.2 s | 148.4 s | 111.7 d |
| 13 | 128.5 s | 128.5 s | 128.5 s | 137.2 s |
| 14 | 30.6 t | 31.1 t | 30.1 t | 36.3 t |
| 15 | 31.5 d | 33.4 d | 32.5 d | 30.6 d |
| 16 | 120.1 s | 119.3 s | 119.8 s | 112.7 s |
| 17 | 160.0 d | 159.8 d | 159.8 d | 152.7 d |
| 18 | 20.9 q | 28.6 q | 12.8 q | 119.0 t |
| 19 | 68.5 d | 207.2 s | 119.8 d | 135.7 d |
| 20 | 47.3 d | 50.6 d | 136.1 s | 45.8 d |
| 21 | 48.5 t | 46.3 t | 54.5 t | 97.1 d |
| 22 | 170.5 s | 167.0 s | 167.1 s | 168.3 s |
| 23 | | | | 55.7 t |
| 24 | | | | 172.9 s |
| 12-OMe | 55.0 q | 56.2 q | 56.2 q | |
| 22-OMe | 53.8 q | 51.7 q | 12-OMe | 51.5 q |
| 24-OMe | | | | 51.8 q |
| 1' | | | | 99.4 d |
| 2' | | | | 74.7 d |
| 3' | | | | 77.7 d |
| 4' | | | | 71.5 d |
| 5' | | | | 78.4 d |
| 6' | | | | 62.5 t |

(Fig. 2). Hence, configurations of alstrostines C–E were same to akummicine, as shown in Fig. 1.

**Figure 2.** Proposed biogenetic pathway of alstrostines C–E skeleton

HRESIMS at *m/z* 603.2553 [M + H]$^+$ defined molecular formula of compound **4** as $C_{30}H_{38}N_2O_{11}$. The IR spectrum of **4** implied the presence of hydroxyls (3424 cm$^{-1}$), double bonds (1627 cm$^{-1}$), and carbonyl groups (1703 cm$^{-1}$). Its UV spectrum showed the characteristic absorption bands of indole alkaloids at 226 and 282 nm[11] in agreement with $^1$H NMR signals at $\delta_H$ 7.39 (1H, d, *J* = 7.8 Hz), 7.28 (1H, d, *J* = 7.8 Hz),

**Table 2.** $^{1}$**H NMR spectroscopic data for compounds 1–4 (1–3 in acetone-*d*$_6$, 4 in methanol-*d*$_4$, *δ* in ppm, *J* in Hz)**

| C | $\delta_H$ (**1**) | $\delta_H$ (**2**) | $\delta_H$ (**3**) | $\delta_H$ (**4**) |
|---|---|---|---|---|
| $N_1$ | | | | 9.65 (1H, br. s) |
| 3 | 3.75 (1H, br. s) | 3.78 (1H, t, 2.6) | 3.88 (1H, t, 2.6) | 4.07 (1H, t, 6.0) |
| 5 | 2.82 (2H, m) | 2.82 (1H, m); 2.98 (1H, m) | 2.79 (1H, m); 2.98 (1H, m) | 3.08 (1H, m); 3.12 (1H, m) |
| 6 | 1.65 (1H, m); 2.82 (1H, m) | 1.68 (1H, m); 2.85 (1H, m) | 3.10 (1H, m); 3.37 (1H, m) | 2.78–2.81 (1H, m); 2.46 (1H, dd, 4.8, 4.5 ) |
| 9 | 6.98 (1H, d, 8.0) | 7.00 (1H, d, 7.9) | 7.02 (1H, d, 7.4) | 7.39 (1H, d, 7.8) |
| 10 | 7.13 (1H, t, 8.0) | 7.16 (1H, t, 7.9) | 7.15 (1H, t, 7.4) | 7.01 (1H, t, 7.8) |
| 11 | 7.03 (1H, d, 8.0) | 7.05 (1H, d, 7.9) | 7.07 (1H, d, 7.4) | 6.96 (1H, t, 7.8) |
| 12 | | | | 7.28 (1H, d, 7.8) |
| 14 | 1.47 (2H, m) | 1.52 (1H, m); 2.11 (1H, m) | 1.50 (1H, m); 2.06 (1H, m) | 1.86 (2H, m) |
| 15 | 3.01 (1H, m) | 3.30 (1H, m) | 3.69 (1H, m) | 3.63 (1H, m) |
| 17 | 9.12 (1H, s) | 9.06 (1H, s) | 9.12 (1H, s) | 7.45 (1H, s) |
| 18 | 1.11 (3H, d, 6.2) | 2.24 (3H, s) | 1.72 (3H, d, 7.2) | 5.75 (2H, m) |
| 19 | 3.44 (1H, m) | | 5.45 (1H, q, 7.2) | 5.36 (1H, m) |
| 20 | 1.65 (1H, m) | 2.90 (1H, m) | | 2.70 (1H, m) |
| 21 | 2.03 (1H, m); 2.80 (1H, m) | 2.53 (1H, m); 2.80 (1H, m) | 3.10 (1H, d, 13.4); 3.37 (1H, d, 13.4) | 5.49 (1H, d, 5.5) |
| 23 | | | | 3.47 (1H, d, 16.5); 3.60 (1H, d, 16.5) |
| 12-OMe | 3.95 (3H, s) | 3.93 (3H, s) | 3.98 (3H,s) | |
| 22-COO<u>Me</u> | 3.67 (3H, s) | 3.60 (3H, s) | 3.67 (3H,s) | 3.68 (3H,s) |
| 24-COO<u>Me</u> | | | | 3.65 (3H,s ) |
| 1' | | | | 4.74 (1H, d, 7.8) |
| 2' | | | | 3.27 (1H, overlap) |
| 3' | | | | 3.43 (1H, overlap) |
| 4' | | | | 3.35 (1H, m) |
| 5' | | | | 3.35 (1H, overlap) |
| 6' | | | | 3.81 (1H, d, 12.8); 3.63 (1H, overlap) |

7.01 (1H, t, $J = 7.8$ Hz), 6.96 (1H, d, $J = 7.8$ Hz). Its $^{13}$C NMR and DEPT spectra displayed six methylenes ($\delta_C$ 119.0, 62.5, 55.7, 44.5, 36.3, 17.5), fourteen methines ($\delta_C$ 152.7, 135.7, 121.7, 119.4, 118.5, 111.7, 99.4, 97.1, 78.4, 77.7, 74.7, 71.5, 58.6, 45.8, 30.6), seven quaternary carbons ($\delta_C$ 136.1, 107.5, 128.2, 137.2, 112.7, 168.3, 172.9), and two methoxyls ($\delta_C$ 51.8, 51.5). Above data were similar to those of strictosidine[12] with exception for additional methylene ($\delta_C$ 55.7, t) and methoxycarbonyl [$\delta_C$ 172.9 (s) and 51.8 (q)] in **4**. In the HMBC spectrum, both H-3 ($\delta_H$ 4.07) and H-5 ($\delta_H$ 3.08 and 3.12) were correlated with $\delta_C$ 55.7 (t), suggesting the methylene was connected with $N_4$. In addition, the methylene protons ($\delta_H$ 3.47 and 3.60) showed correlations to 172.9 (s), indicating **4** was strictosidine methyl $N^4$-acetate.

All alkaloids **1**–**17** were tested for their ability to prevent the cytopathic effects of cancer in breast cancer SK-BR-3, hepatocellular carcinoma SMMC-7721, human myeloid leukemia HL-60, pancreatic cancer PANC-1, and lung cancer A-549 cells, and their cytotoxicity was measured in parallel with the determination of antitumor activity using cisplatin as the positive control. Unfortunately, none of them showed positive activity ($IC_{50} > 40$ $\mu$M).

## Experimental Section

**General Experimental Procedures.** Optical rotations were measured with a Horiba SEAP-300 spectropolarimeter. UV spectra were recorded on a Shimadzu double-beam 210A spectrophotometer. IR (KBr) spectra were obtained on Bio-Rac. FTS-135 infrared spectrophotometer. $^{1}$H, $^{13}$C and 2D NMR spectra were recorded on a AM-400 and DRX-500 MHz NMR spectrometer with TMS as an internal standard. MS data were obtained on an API Qstar Pulsar I spectrometer. Silica gel (200–300 mesh) for column chromatography (CC) and $GF_{254}$ for TLC were obtained from Qingdao Marine Chemical Factory, Qingdao, China and sprayed with Dragdorff' reagent. C18 silica gel (20–45 $\mu$m) was bought from Fuji Chemical Ltd., Japan. MPLC was employed Buchi pumps system coupled with glass column (15 × 230 and 26 × 460 mm, respectively, C18 silica gel). HPLC was performed using Waters 600 pumps coupled with analytical and semipreparative sunfire C18 columns (150 × 4.6 and 150 × 10 mm, respectively). The HPLC system employed a Waters 2996 photodiode array detector and a Waters fraction collector II.

**Plant Material.** *Alstonia rostrata* C. E. C. Fischer was colleted in Apr. 2010 in Simao of Yunnan Province, China, and identified by Dr. Chun-Xia Zeng. Voucher specimen (Cai100613) deposited in the State Key Laboratory of Phytochemistry and Plant Resources in West China, Kunming Institute of Botany, the Chinese Academy of Sciences.

**Extraction and Isolation.** Air-dried leaves and twigs (8.0 kg) of *A. rostrata* was crushed and extracted with EtOH (20 L × 3). After removal of the EtOH under reduced pressure, the residue was dissolved in 1% HCl, and partitioned with EtOAc for three times. The acidic solution was subsequently basified using ammonia water to pH 8~9, and partitioned with EtOAc for three times, affording a two-phase mixture including the aqueous phase, EtOAc/organic phase (total alkaloids). The total alkaloid fraction (78 g) was collected and then dissolved in MeOH, and was subjected to column chromatography over silica gel eluting with $CHCl_3$-MeOH [from $CHCl_3$ to $CHCl_3$-MeOH (1:1)] to afford six fractions (I–VI) according to differences in composition monitored by TLC plate after spraying with dragendorff's reagent. Fraction I was further purified by column chromatography on silica gel (35 g) using petroleum ether-$Me_2CO$ (9:1) to give **17** (220 mg). Fraction II (4.5 g) was subjected to MPLC over $RP_{18}$ gel (52 g) column, eluting with MeOH-$H_2O$ (from 2:8 to 9:1) to afford six subfractions (II-1~II-6)**.** Fraction II-2 (2.5 g) was further purified by column chromatography on silica gel (35 g) using $CHCl_3$-$Me_2CO$ (9:1~4:1) to give **2** (220 mg), II-2-2 (45 mg) and II-2-3 (1.6 g). II-2-2 was seprarted by semipreparative reversed-phase C18 HPLC on Xterra column with gradient flow from 35% to 65% aquous methanol to affort pure compound **1** (30 mg). II-2-2-3(100 mg) was subjected to MPLC over C18 gel (26 g) column, eluting with MeOH-$H_2O$ (from 2:3 to 7:3) to afford pure compound **6** (300 mg) and **7** (50 mg). Fraction II-5 (300 mg) was further purified by semi-preparative column with gradient flow from 50 to 75% aquous methanol to give **3** (40 mg). Same semipreparative column with gradient flow from 40 to 60% aquous methanol was used to separate combination of subfractions II-6 (70 mg) .This technique afforded **5** (25 mg). Fr.III (1.5 g) was subjected on C18 silica gel CC and eluted with 30–80% aquous methanol to give 6 subfractions III-1~6. Fraction III-3 (200 mg) was loaded on silica gel (20 g) CC eluting by petroleum $CHCl_3$-$Me_2CO$ (from 6:1 to 4:1) to obtain **8** (18 mg). Fraction III-5 (50 mg) were subjected to a C18 semipreparative column and eluted with 50–80% aquous methanol to afford compound **4** (5 mg). Fraction III-6 (800 mg) were subjected to a C18 semipreparative column and eluted with 40–80% aquous methanol to afford **9** (13 mg) and **12** (40 mg). Fr.IV (6.5g) was subjected on C.C. over Rp-18 silica gel (160 g) and eluted with 40–100% methanol to affort subfractions IV-1~5. Fraction IV-1 (1.15 g) was rechromatographed on a Si gel (30 g) column and eluted with petroleum $CHCl_3$-$Me_2CO$ (from 3:1 to 1:1) to give **10** (11 mg) and **16** (70 mg)**.** Fraction IV-3 (1.9 g) was rechromatographed on a C18 Si gel (50 g) column and eluted with 50% aquous methnal to give **13** (28 mg) and **14** (36 mg)**.** Fraction IV-5 **(**50mg**)** was further purified by column chromatography on silica gel (5 g) using $CHCl_3$-$Me_2CO$ (2:1~1:1) to give alkaloid **15** (4 mg). Alkaloid **11** (600 mg) is acrystal from fraction V.

**Alstrostine C (1):** $C_{22}H_{26}N_2O_5$, yellow powder. $[\alpha]_D^{18}$ +47 (*c* 0.16, MeOH). UV (MeOH): 214 (3.95), 275 (3.26), 326 (3.02); IR (KBr): 3453, 2939, 1705, 1646, 1366 $cm^{-1}$; $^1H$ NMR (400 MHz) and $^{13}C$ NMR (100 MHz), see Tables 1 and 2, respectively. Positive ESIMS *m/z*: 399 $[M + H]^+$, HRESIMS *m/z*: 399.1915 $[M + H]^+$ (calcd. for $C_{22}H_{27}N_2O_5$ 399.1919).

**Alstrostine D (2):** $C_{22}H_{24}N_2O_5$, yellow powder. $[\alpha]_D^{18}$ +41.8 (*c* 0.15, $CH_3OH$); UV (MeOH): 211 (4.03), 274 (3.27), 326 (3.11); IR (KBr): 3432, 2928, 1709, 1694, 1644 $cm^{-1}$; $^1H$ (400 MHz) and $^{13}C$ NMR (100 MHz), see Tables 1 and 2, respectively. Positive ESIMS *m/z*: 397 $[M + H]^+$; HRESIMS *m/z*: 397.1756 $[M + H]^+$ (calcd. for $C_{22}H_{25}N_2O_5$ 397.1763).

**Alstrostine E (3):** $C_{22}H_{24}N_2O_4$, yellow powder. $[\alpha]_D^{18}$ +193 (*c* 0.11, MeOH); UV (MeOH): 213 (3.93), 274 (3.15), 326 (3.03) nm; IR (KBr): 3432, 2946, 1696, 1654, 1452, 1359 $cm^{-1}$; $^1H$ NMR (400 MHz) and $^{13}C$ NMR (100 MHz), see Tables 1 and 2, respectively. Positive ESIMS *m/z*: 381 $[M + H]^+$, HRESIMS *m/z*: 381.1820 $[M + H]^+$ (calcd. for $C_{22}H_{25}N_2O_4$ 381.1814).

**Alstrostine F (4):** $C_{30}H_{38}N_2O_{11}$, white powder. $[\alpha]_D^{19}$ –70 (*c* 0.11, MeOH). UV (MeOH): 226 (3.96), 282 (3.14) nm; IR (KBr): 3424, 2924, 1703, 1627, 1075 $cm^{-1}$; $^1H$ NMR (500 MHz) and $^{13}C$ NMR (100 MHz), see Tables 1 and 2, respectively. Positive ESIMS *m/z*: 603 $[M + H]^+$, HRESIMS *m/z*: 603.2536 $[M + H]^+$ (calcd. for $C_{30}H_{39}N_2O_{11}$ 603.2553).

**Cytotoxicity Assay.** Five human cancer cell lines, breast cancer SK-BR-3, hepatocellular carcinoma SMMC-7721, human myeloid leukemia HL-60, pancreatic cancer PANC-1, and lung cancer A-549 cells, were used in the cytotoxic assay. All the cells were cultured in RPMI-1640 or DMEM medium (Hyclone, USA), supplemented with 10% fetal bovine serum (Hyclone, USA) in 5% $CO_2$ at 37 °C. The cytotoxicity assay was performed according to the MTT (3-(4,5-dimethylthiazol-2-yl)-2,5-diphenyl tetrazolium bromide) method in 96-well microplates.[13] Briefly, 100 $\mu$L adherent cells were seeded into each well of 96-well cell culture plates and allowed to adhere for 12 h before drug addition, while suspended cells were seeded just before drug addition with initial density of $1 \times 10^5$ cells/ml. Each tumor cell line was exposed to the test compound at concentrations of 0.0625, 0.32, 1.6, 8, and 40 $\mu$m in triplicates for 48 h, with cisplatin (sigma, USA) as the positive control. After compound treatment, cell viability was detected and cell growth curve was graphed. $IC_{50}$ value was calculated by Reed and Muench's method.[14]

## Electronic Supplementary Material

Supplementary material is available in the online version of this article at http://dx.doi.org/ 10.1007/s13659-012-0019-y and is accessible for authorized users.

## Acknowledgments

This project was supported by the National Natural Science Foundation of China (21172225, 31170334), the 973 Program of Ministry of Science and Technology of China (2009CB522300), the *XiBuZhiGuang* Project of Chinese Academy of Sciences, and the Young Academic and Technical Leader Raising Foundation of Yunnan Province (No. 2010CI049).

**Open Access** This article is distributed under the terms of the Creative Commons Attribution License which permits any use, distribution, and reproduction in any medium, provided the original author(s) and source are credited.

## Electronic supplementary material

Supplementary material, approximately 799 KB.
