# Supplementary material for: Monoterpenoid indole alkaloids from Alstonia rostrata
Source: Nat Prod Bioprospect. 2012 Apr 19;2(3):121–5. doi: 10.1007/s13659-012-0019-y (PMC4131594; doi:10.1007/s13659-012-0019-y)

Electronic Supplementary Material

Monoterpenoid indole alkaloids from *Alstonia rostrata*

Mei-Fen BAO,[a,c] Chun-Xia ZENG,[b] Yan QU,[a] Ling-Mei KONG,[a] Ya-Ping LIU,[a] Xiang-Hai CAI,[a,]* and Xiao-Dong LUO[a,]*

[a]State Key Laboratory of Phytochemistry and Plant Resources in West China, Kunming Institute of Botany, Chinese Academy of Sciences, Kunming 650201, China

[b]Southwest China Germplasm Bank of Wild Species, Kunming Institute of Botany, Chinese Academy of Sciences, Kunming 650201, China

[c]Graduate University of Chinese Academy of Sciences, Beijing 100049, China

Received 5 March 2012; Accepted 9 April 2012

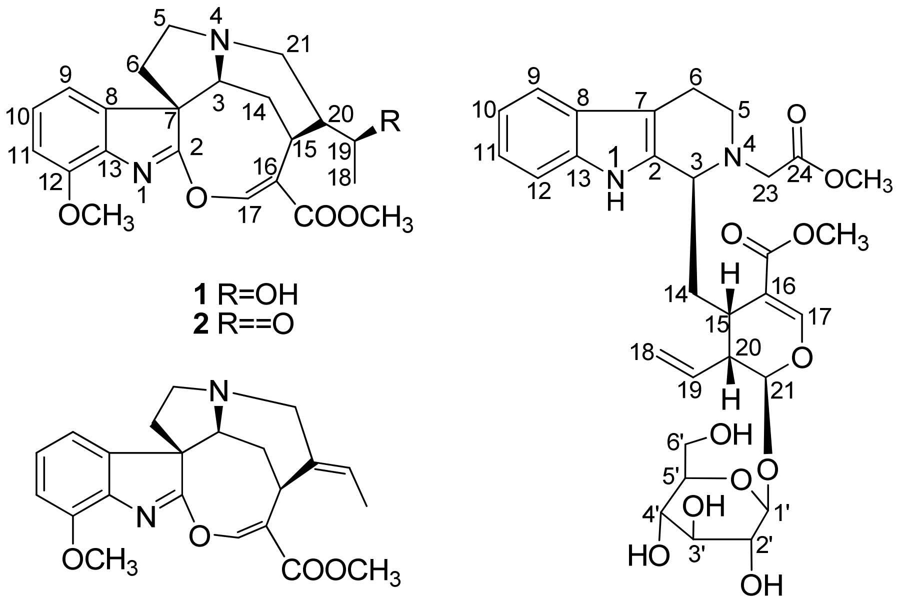

3 **4**

Structures of compounds **1**–**4**

*To whom correspondence should be addressed. E-mail: xhcai@mail.kib.ac.cn (X.H. Cai); xdluo@mail.kib.ac.cn (X.D. Luo).

Figure 1. ^{1}H NMR spectrum of alstrostine C (**1**)

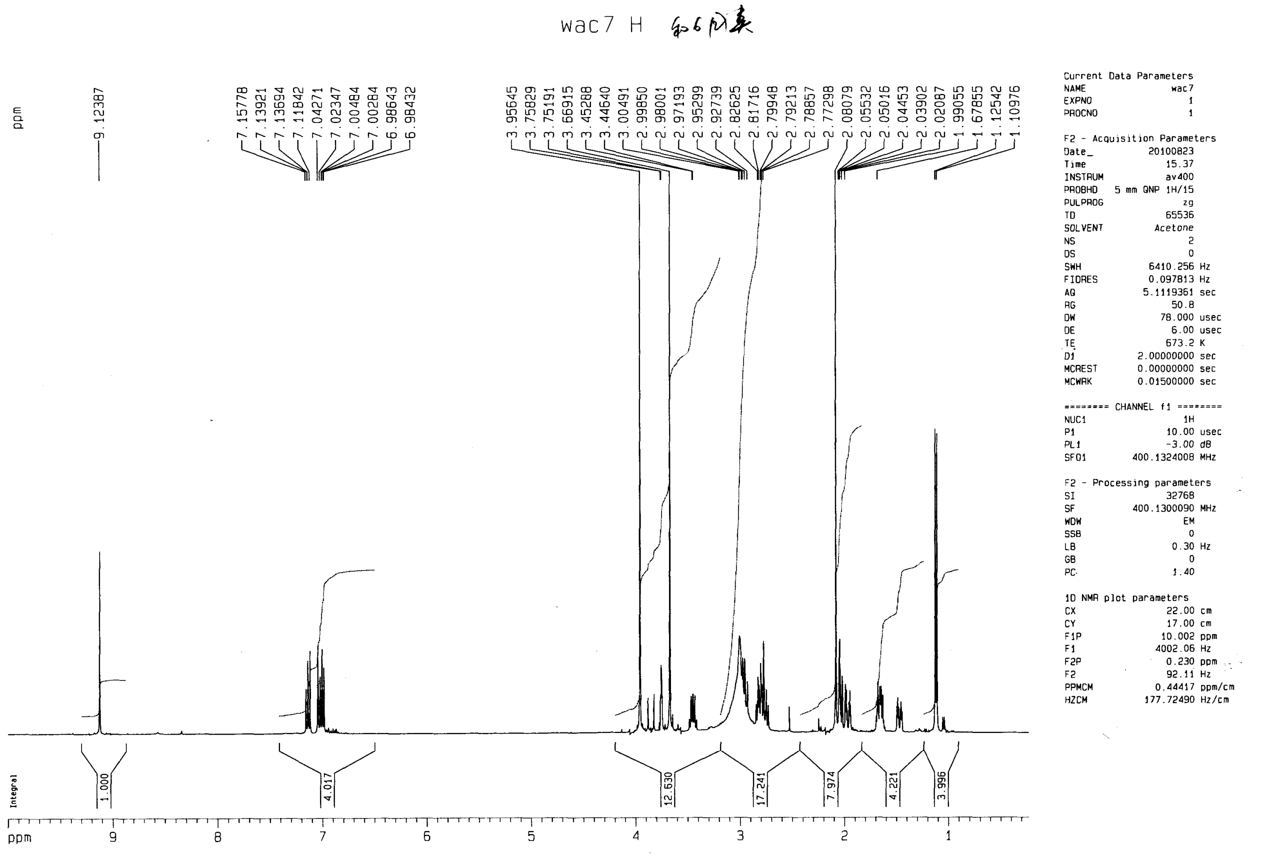

Figure 2. ^{13}C NMR spectrum of alstrostine C (**1**)

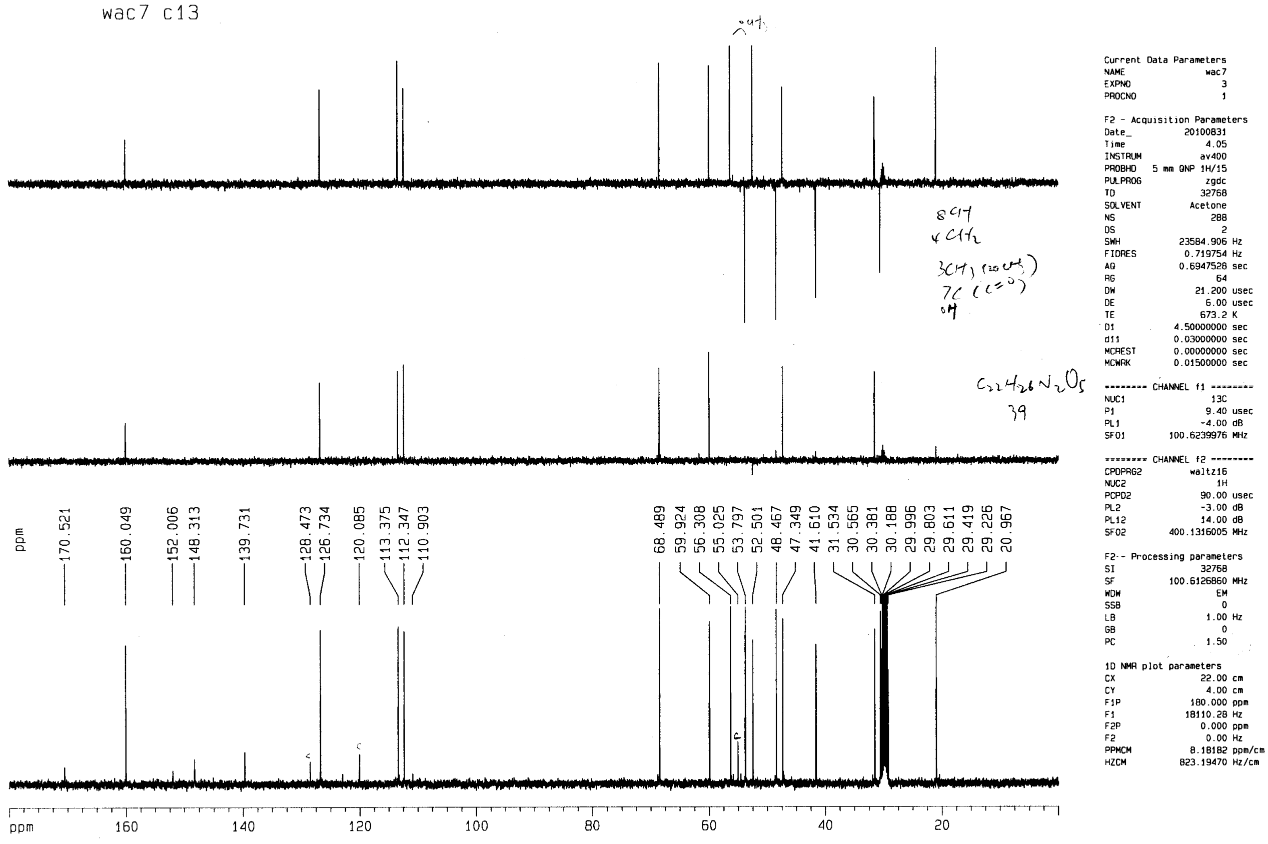

Figure 3. HSQC spectrum of alstrostine C (**1**)

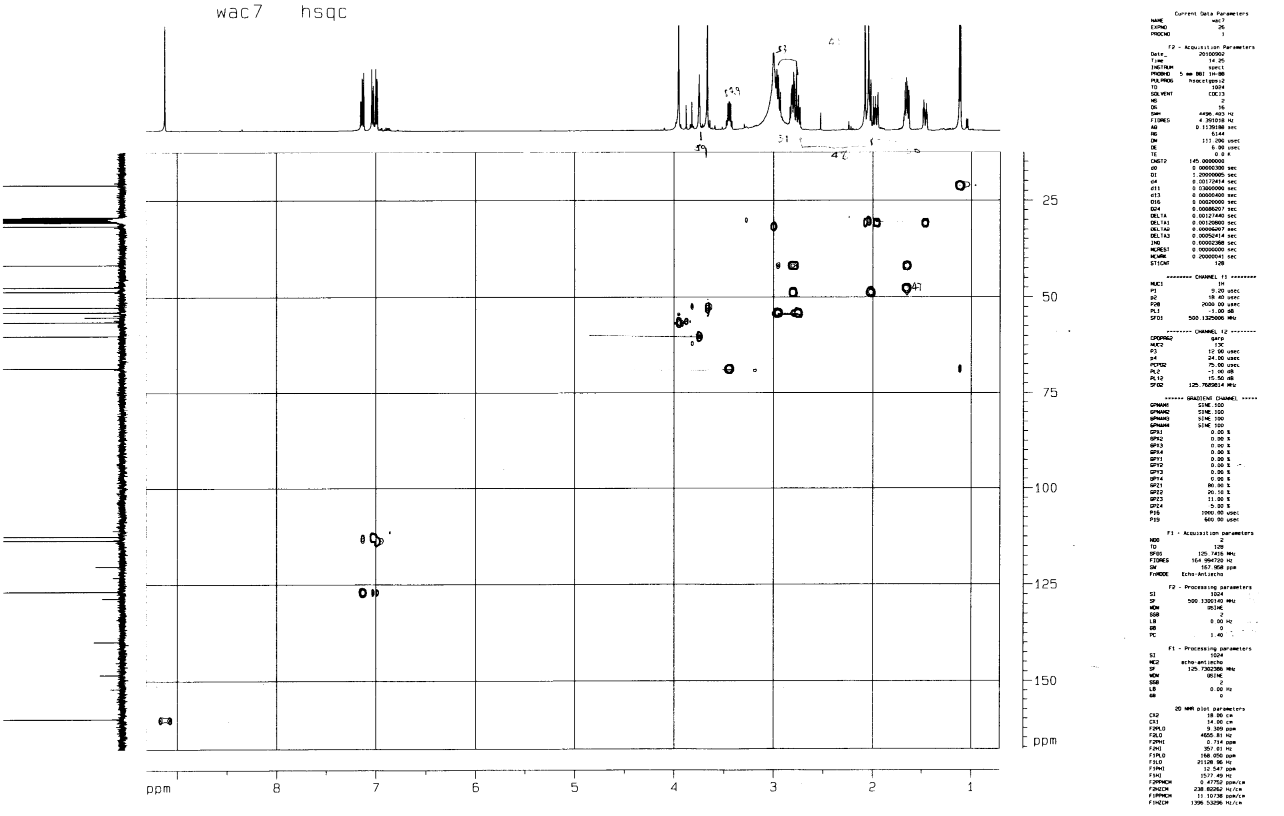

Figure 4. HMBC spectrum of alstrostine C (**1**)

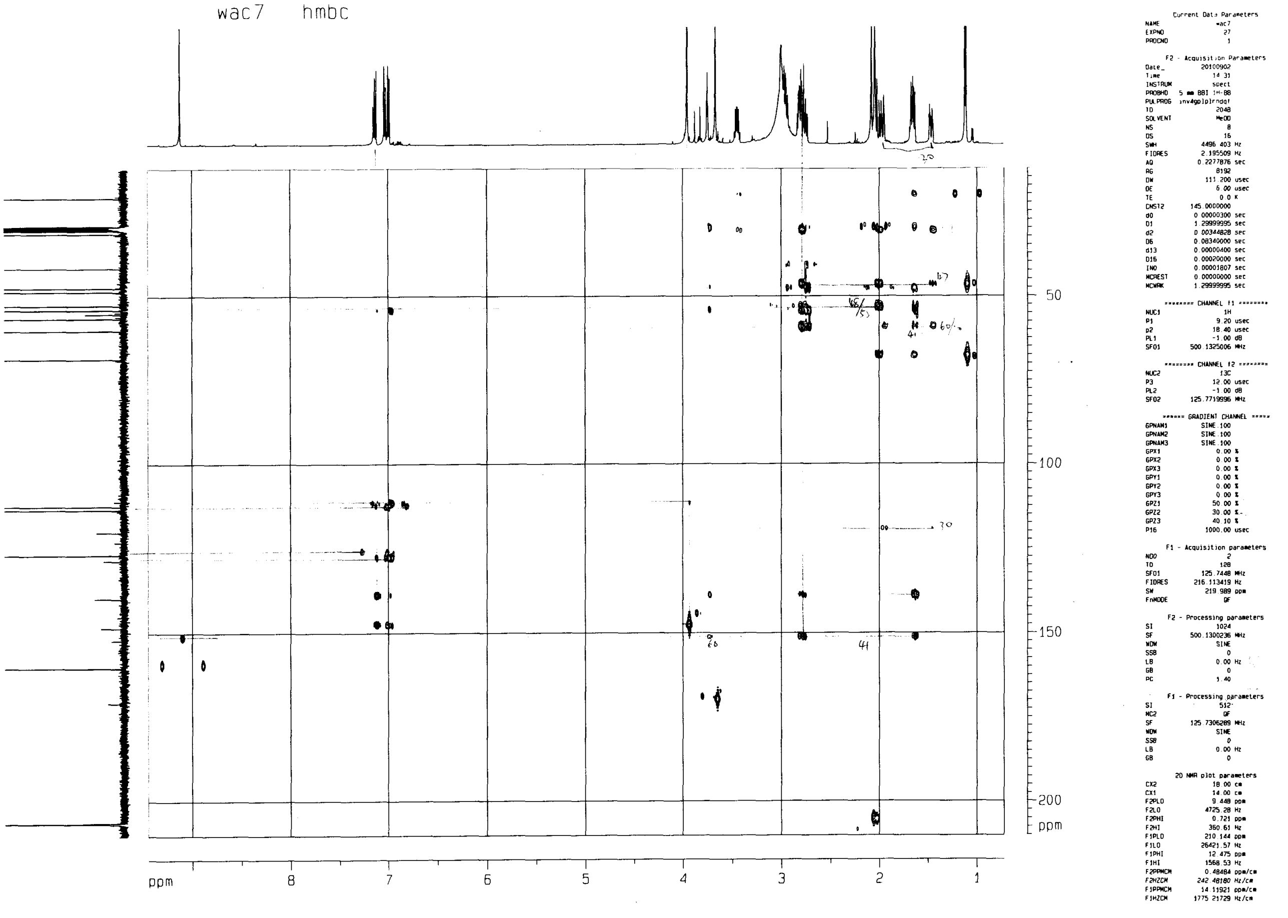

Figure 5. ROESY spectrum of alstrostine C (**1**)

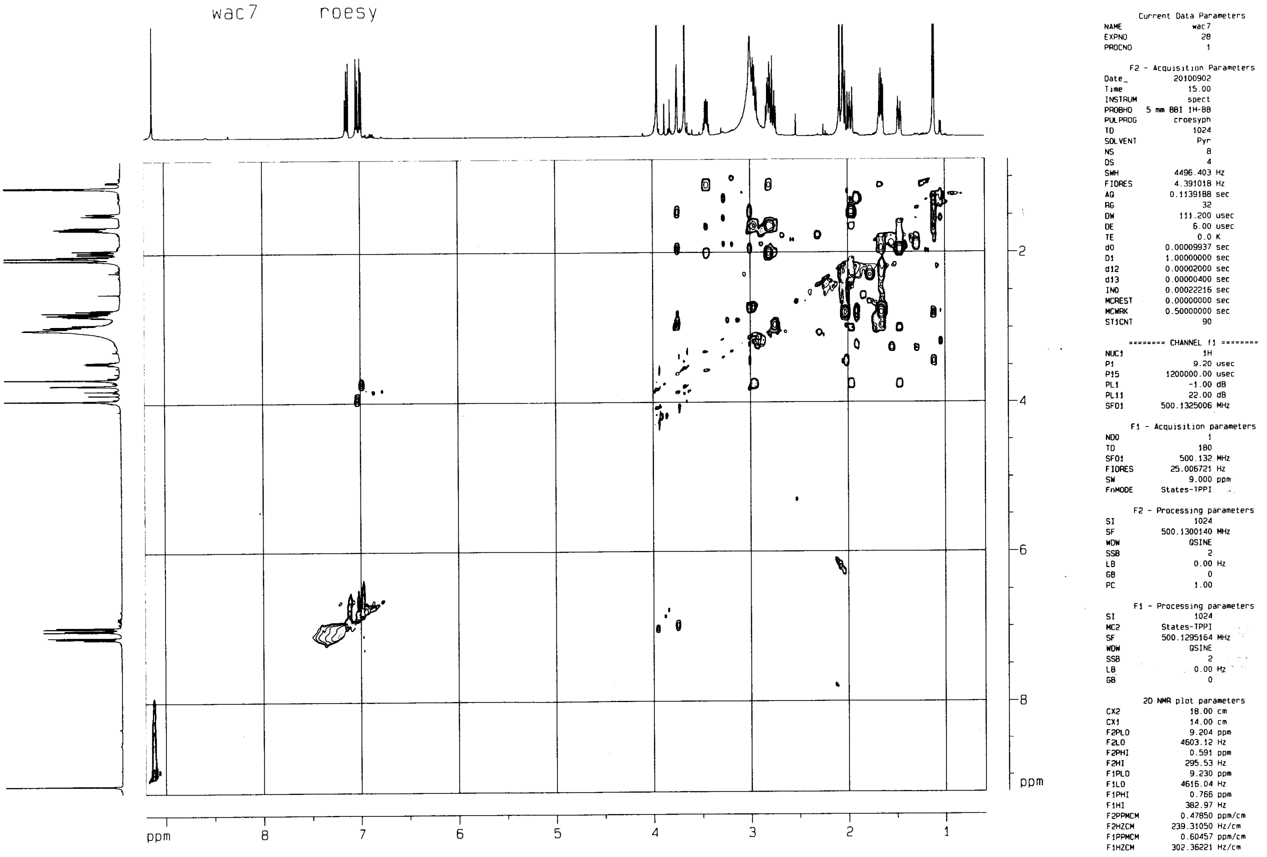

Figure 6. ^{1}H NMR spectrum of alstrostine D (**2**)

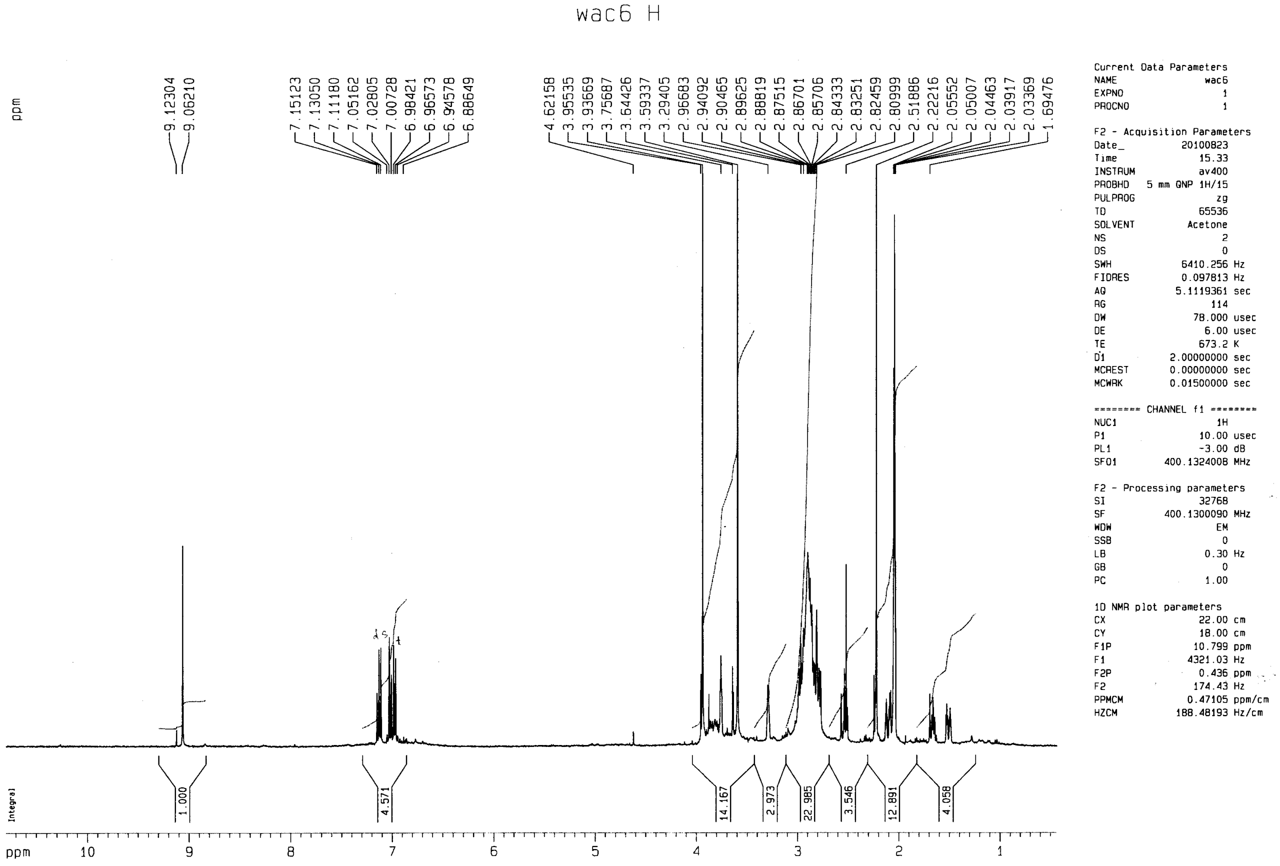

Figure 7. ^{13}C NMR spectrum of alstrostine D (**2**)

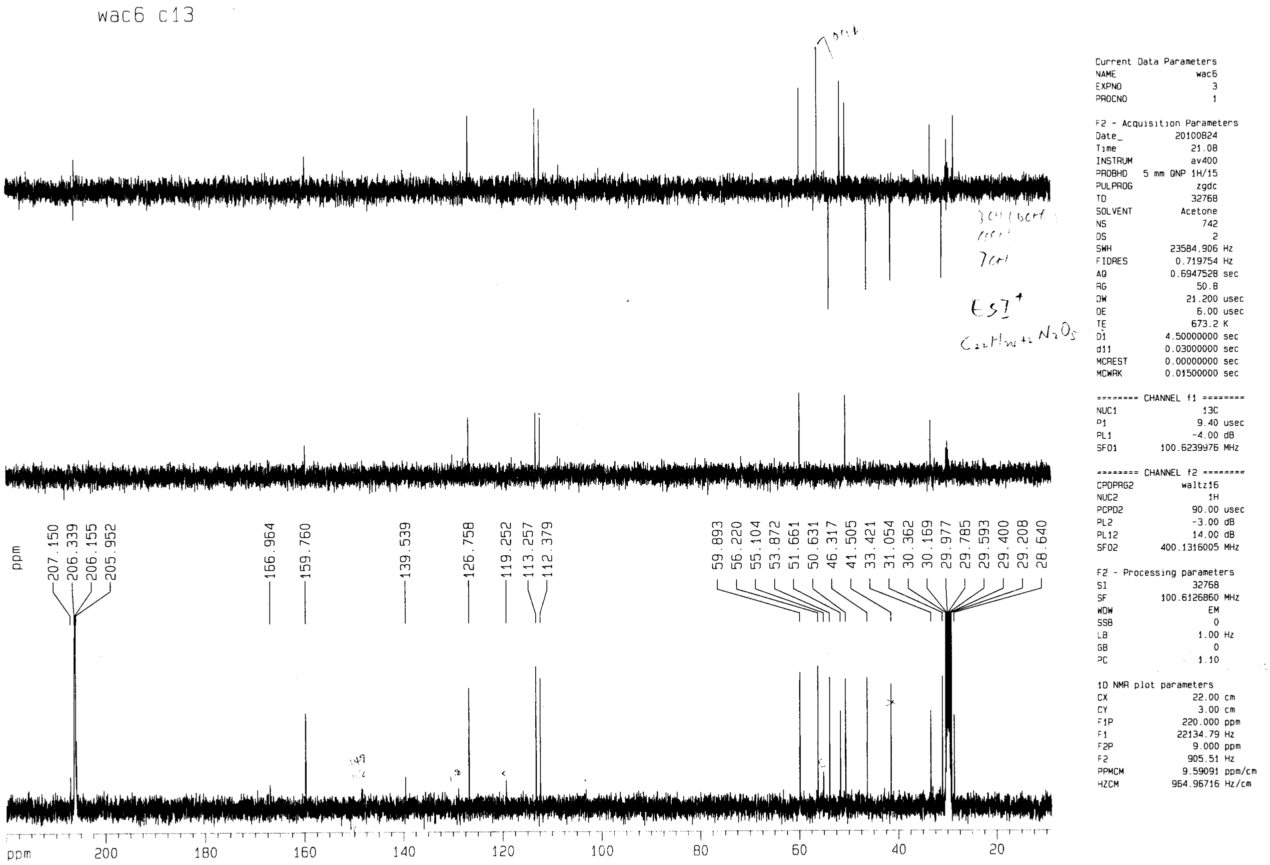

Figure 8. HSQC spectrum of alstrostine D (**2**)

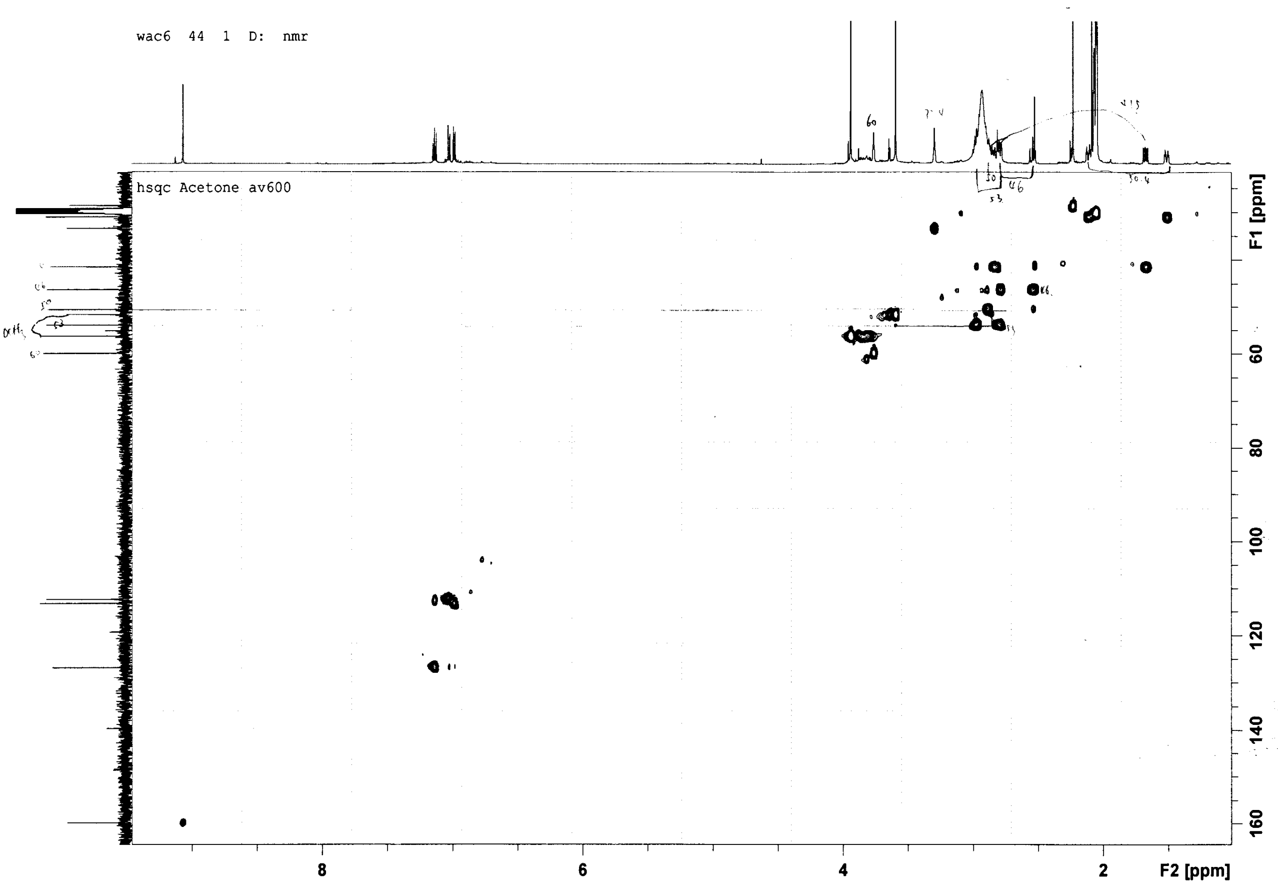

Figure 9. HMBC spectrum of alstrostine D (**2**)

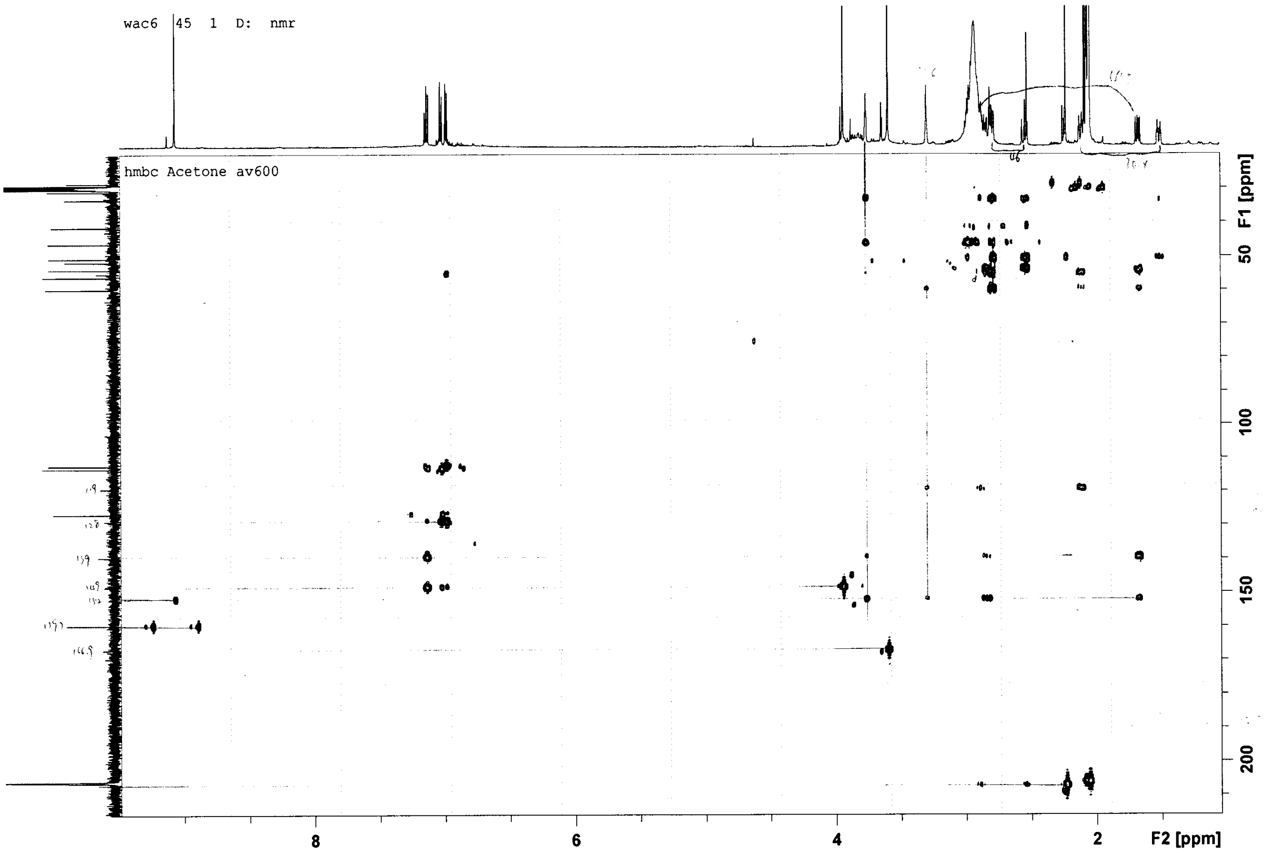

Figure 10. ROESY spectrum of alstrostine D (**2**)

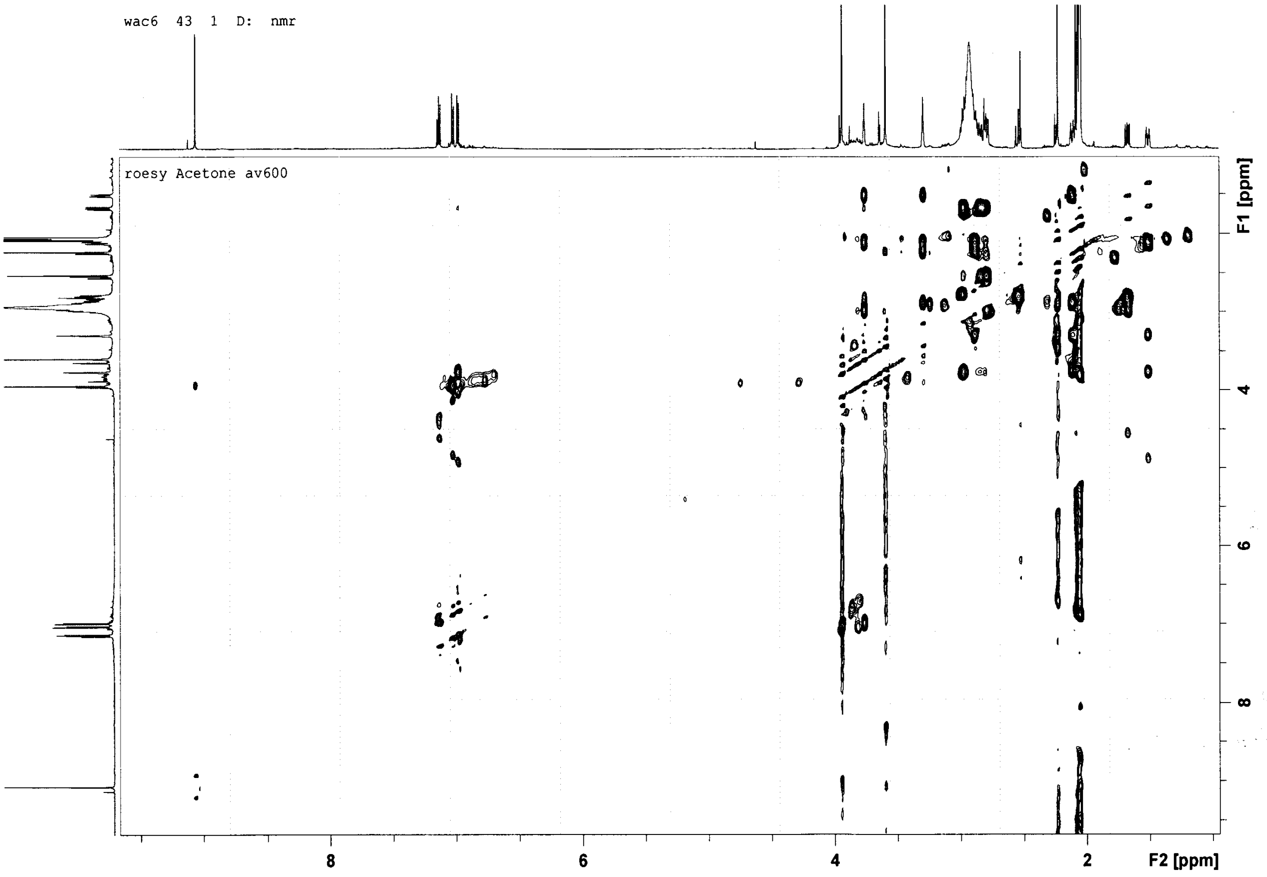

Figure 11. ^{1}H NMR spectrum of alstrostine E (**3**)

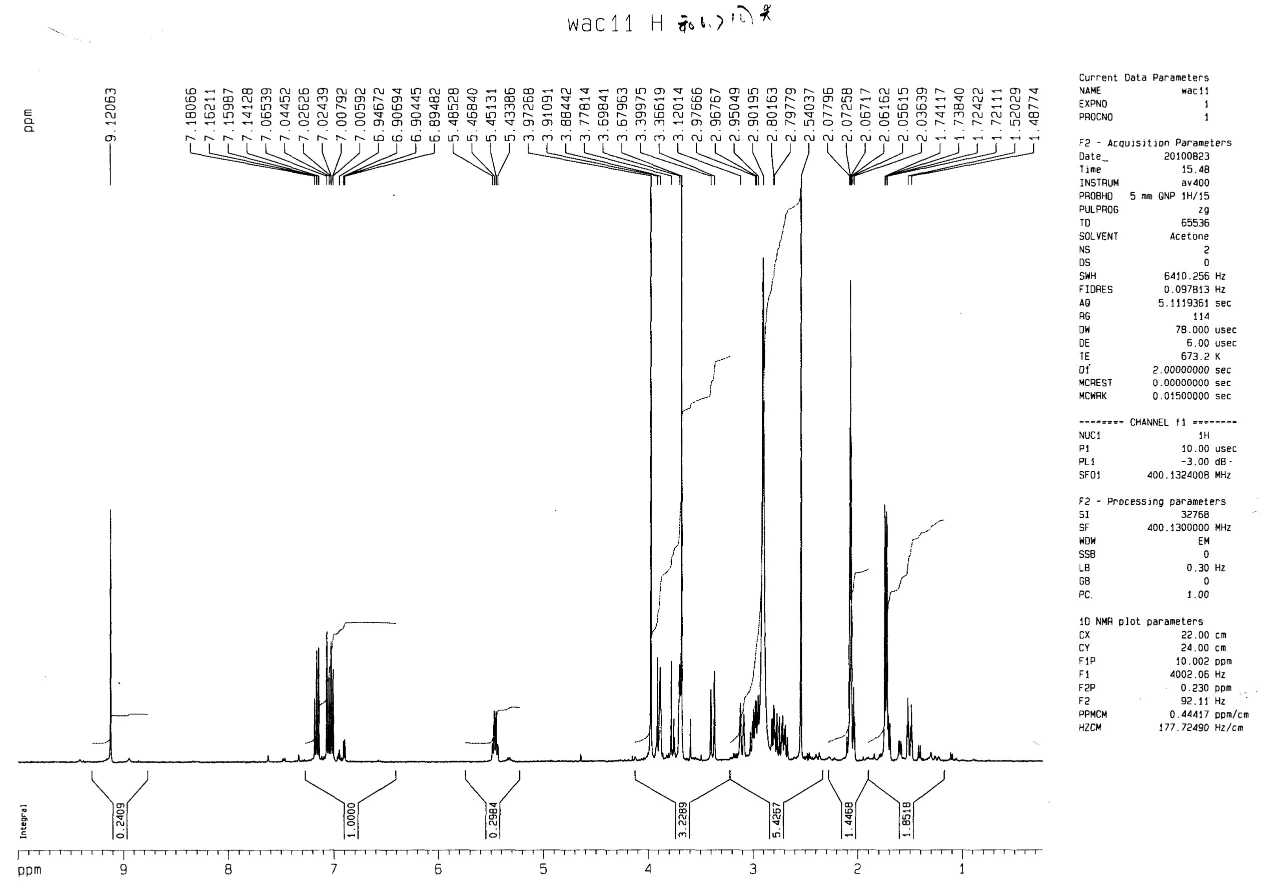

Figure 12. ^{13}C NMR spectrum of alstrostine E (**3**)

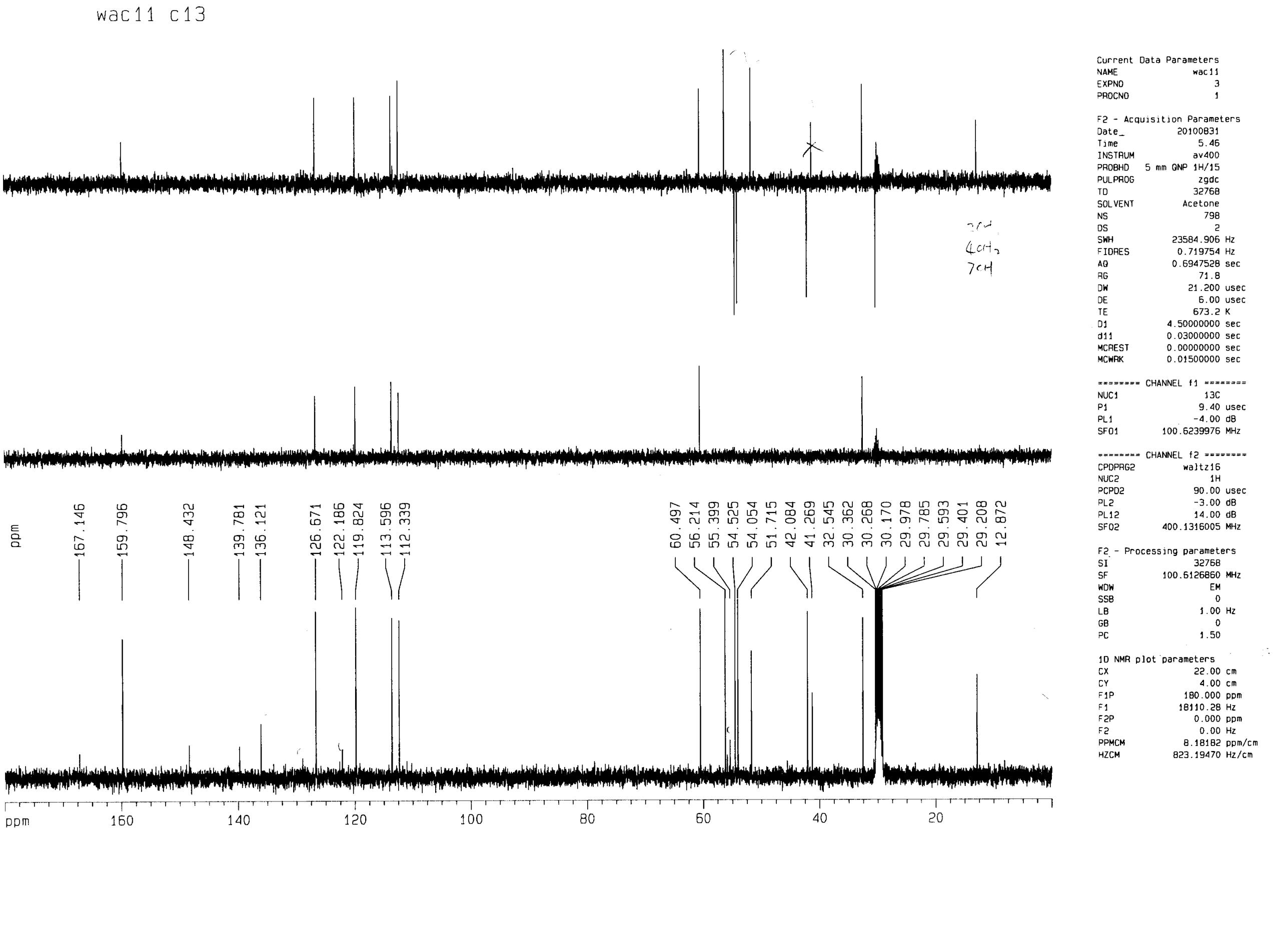

Figure 13. HSQC spectrum of alstrostine E (**3**)

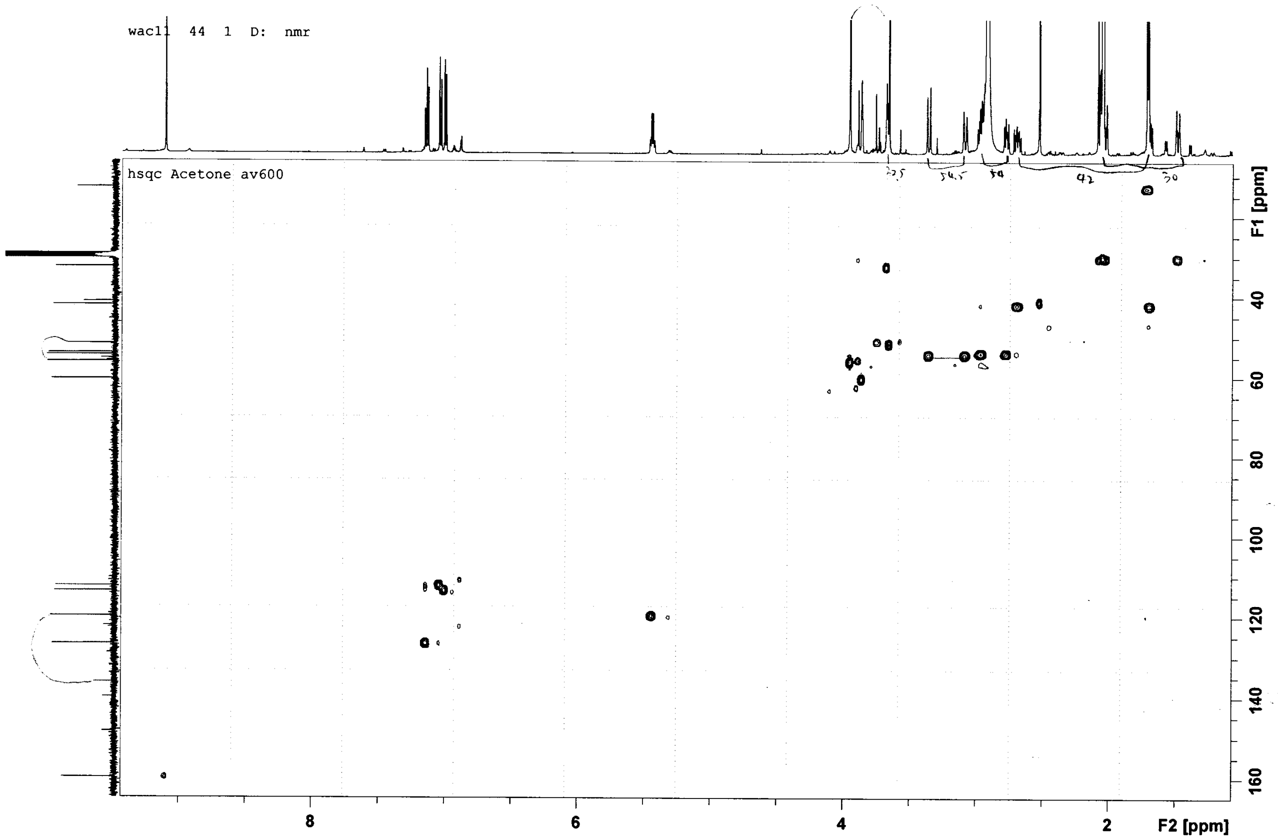

Figure 14. HMBC spectrum of alstrostine E (**3**)

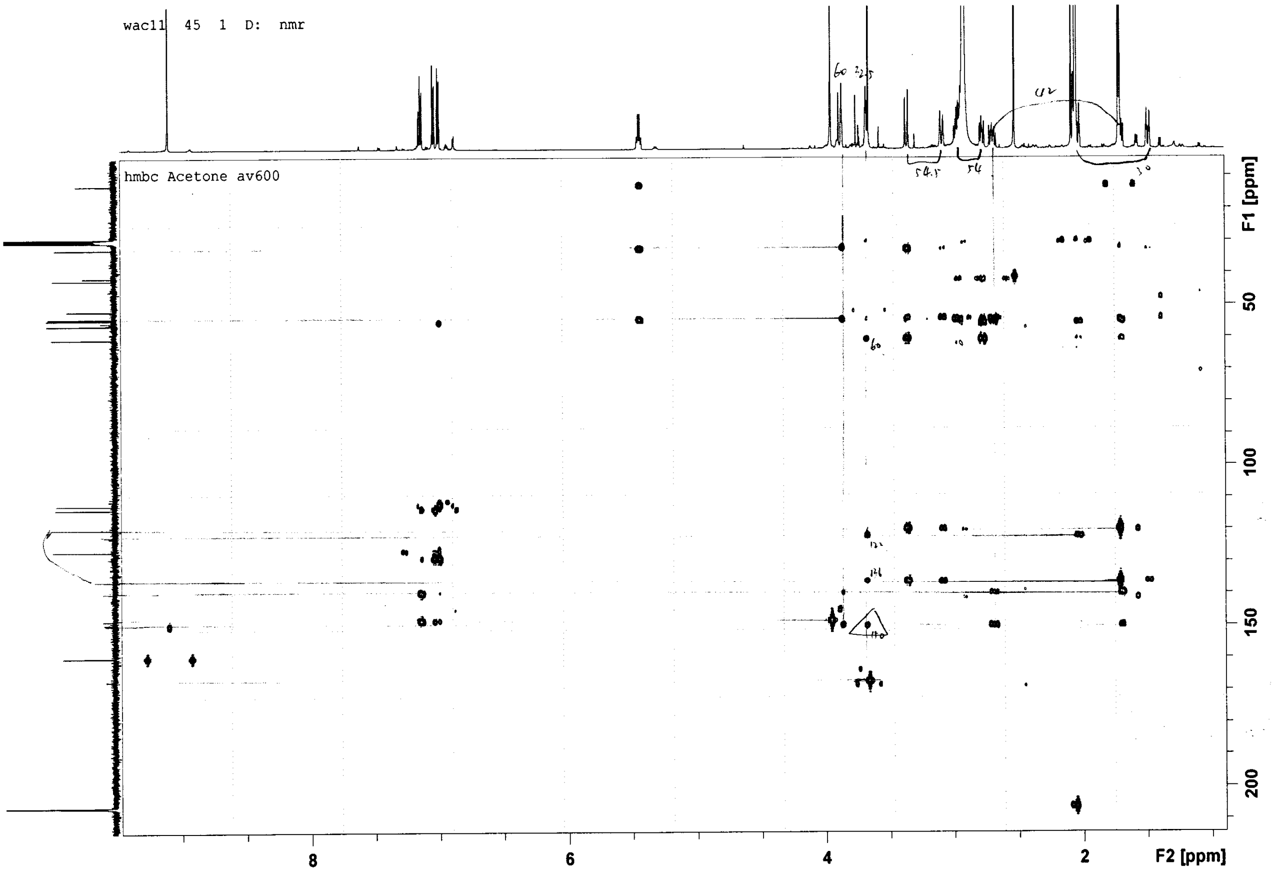

Figure 15. ROESY spectrum of alstrostine E (**3**)

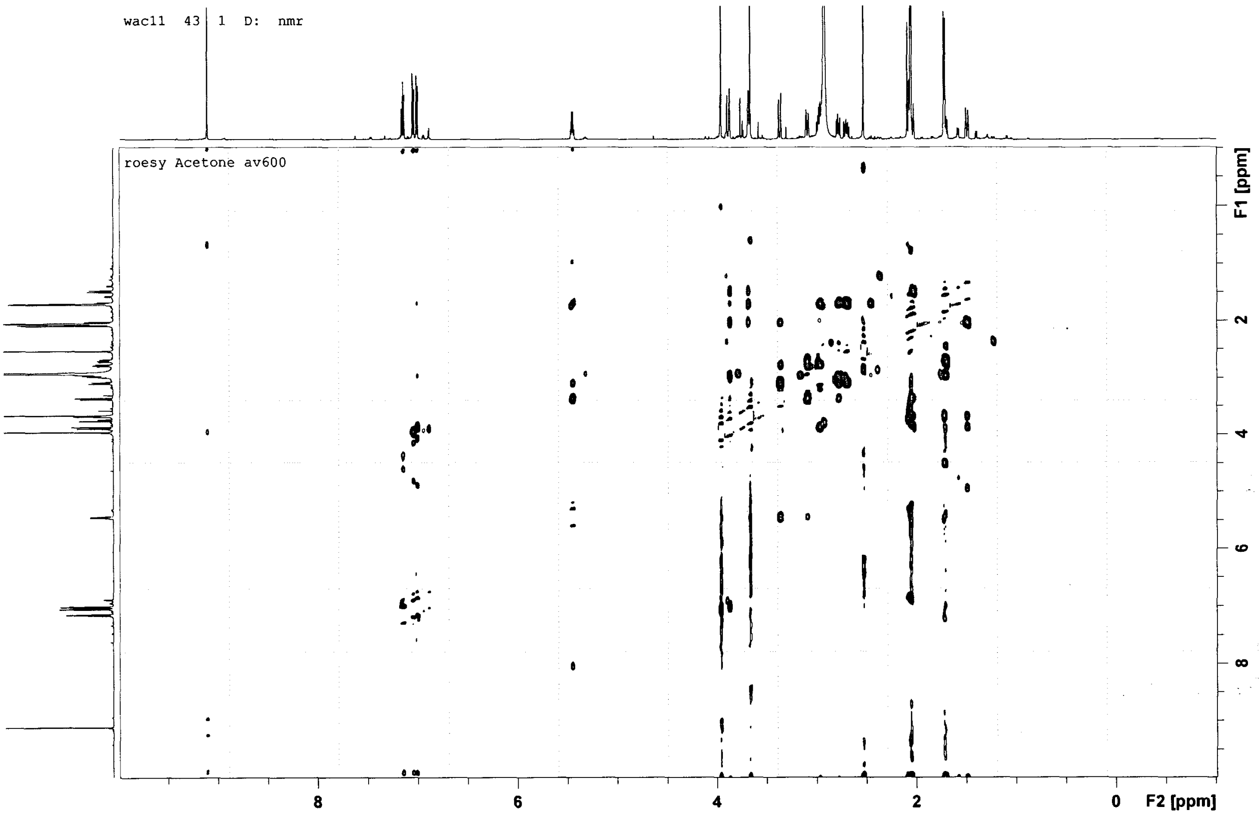

Figure 16. ^{1}H NMR spectrum of alstrostine F (**4**)

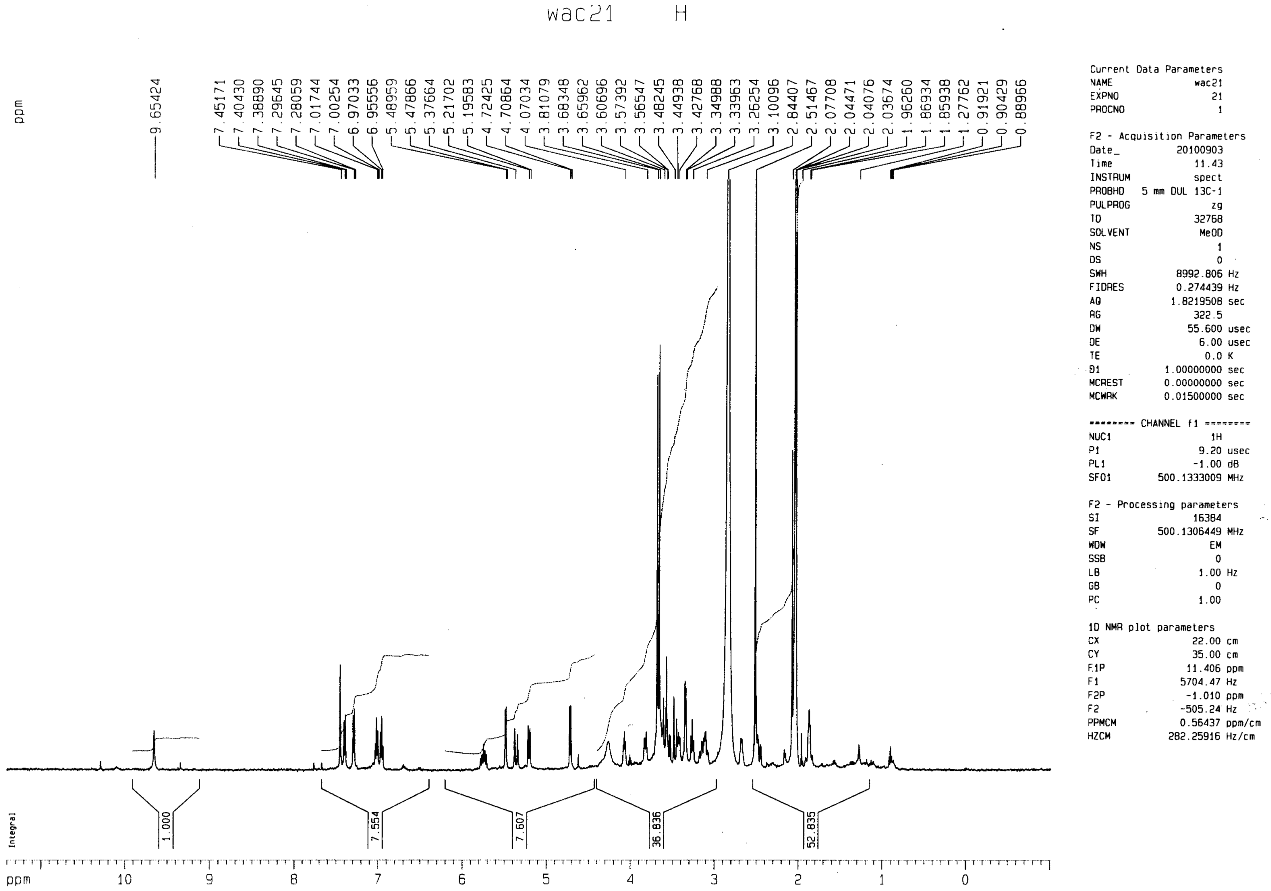

Figure 17. ^{13}C NMR spectrum of alstrostine F (**4**)

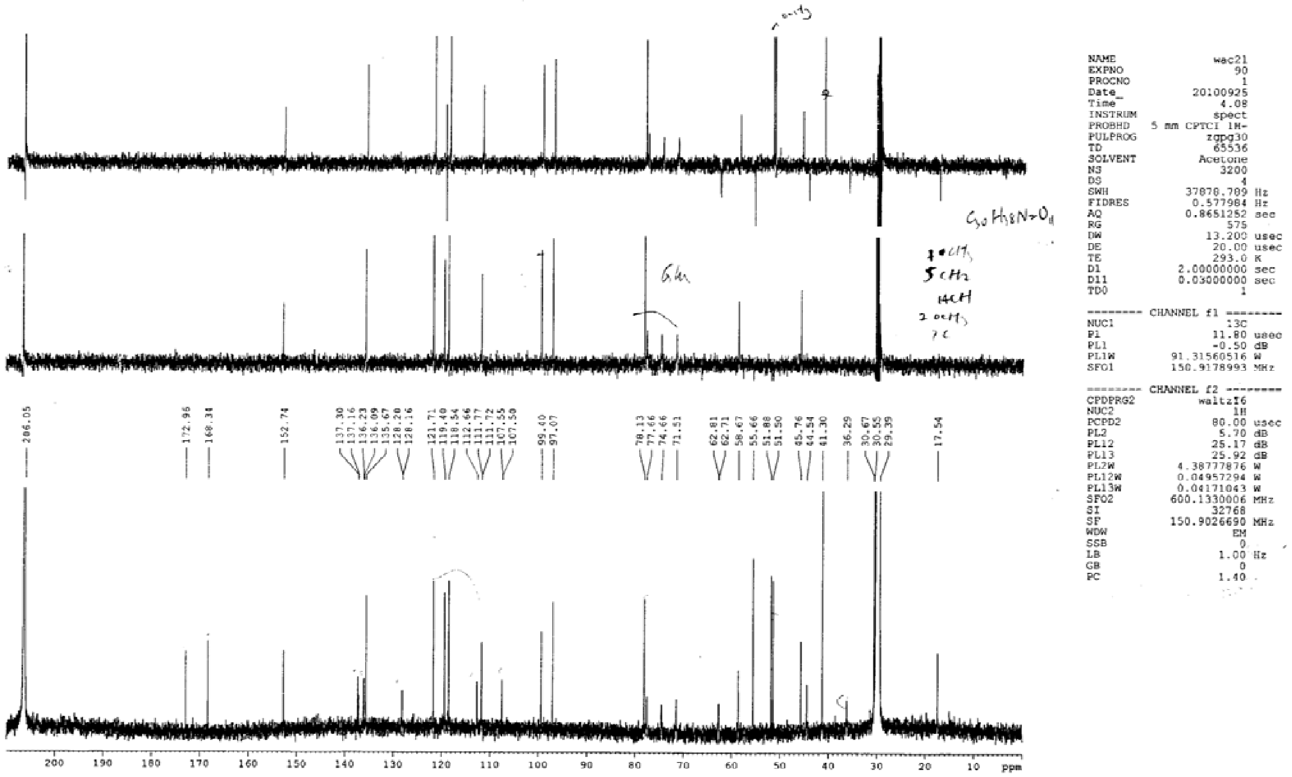

Figure 18. HSQC spectrum of alstrostine F (**4**)

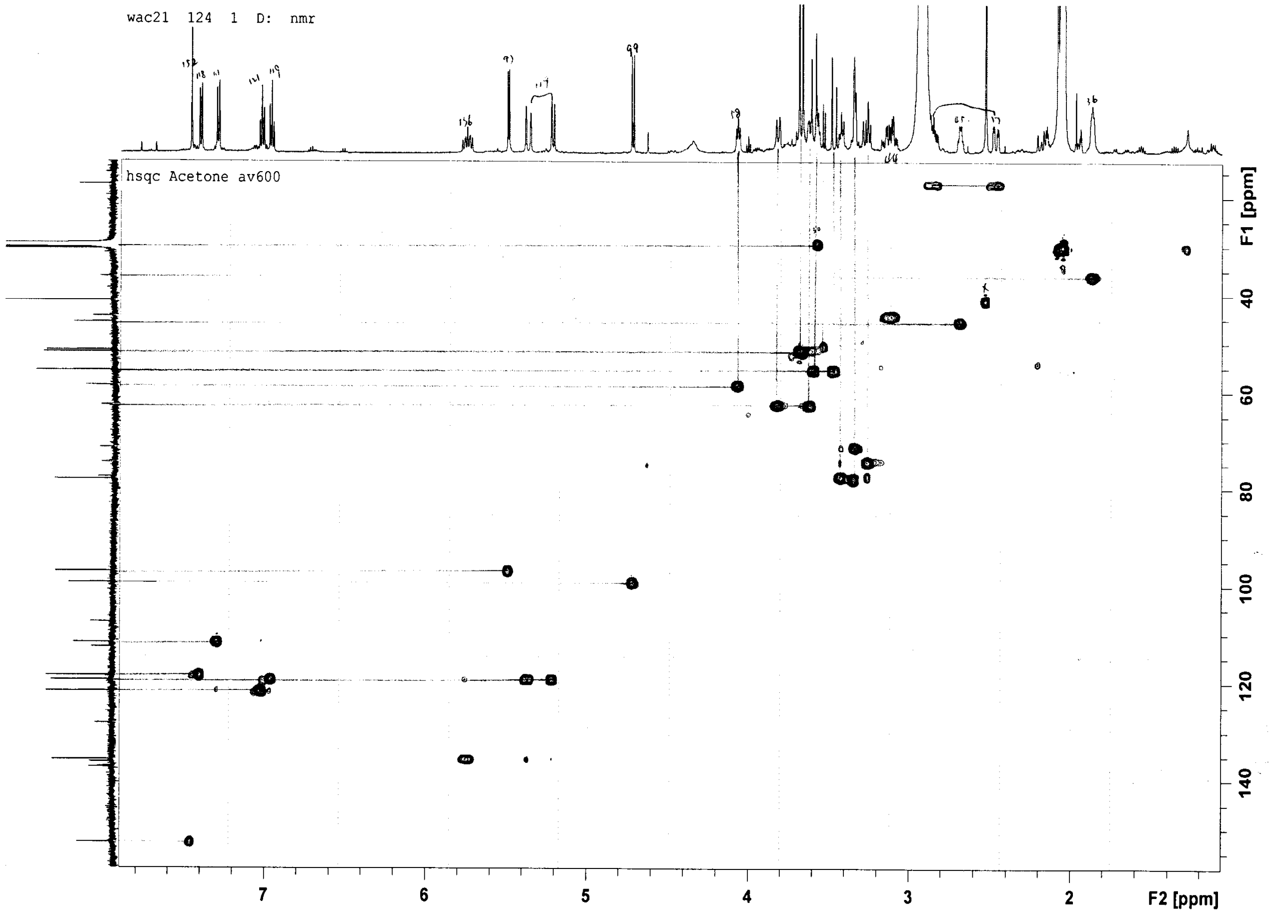

Figure 19. HMBC spectrum of alstrostine F (**4**)

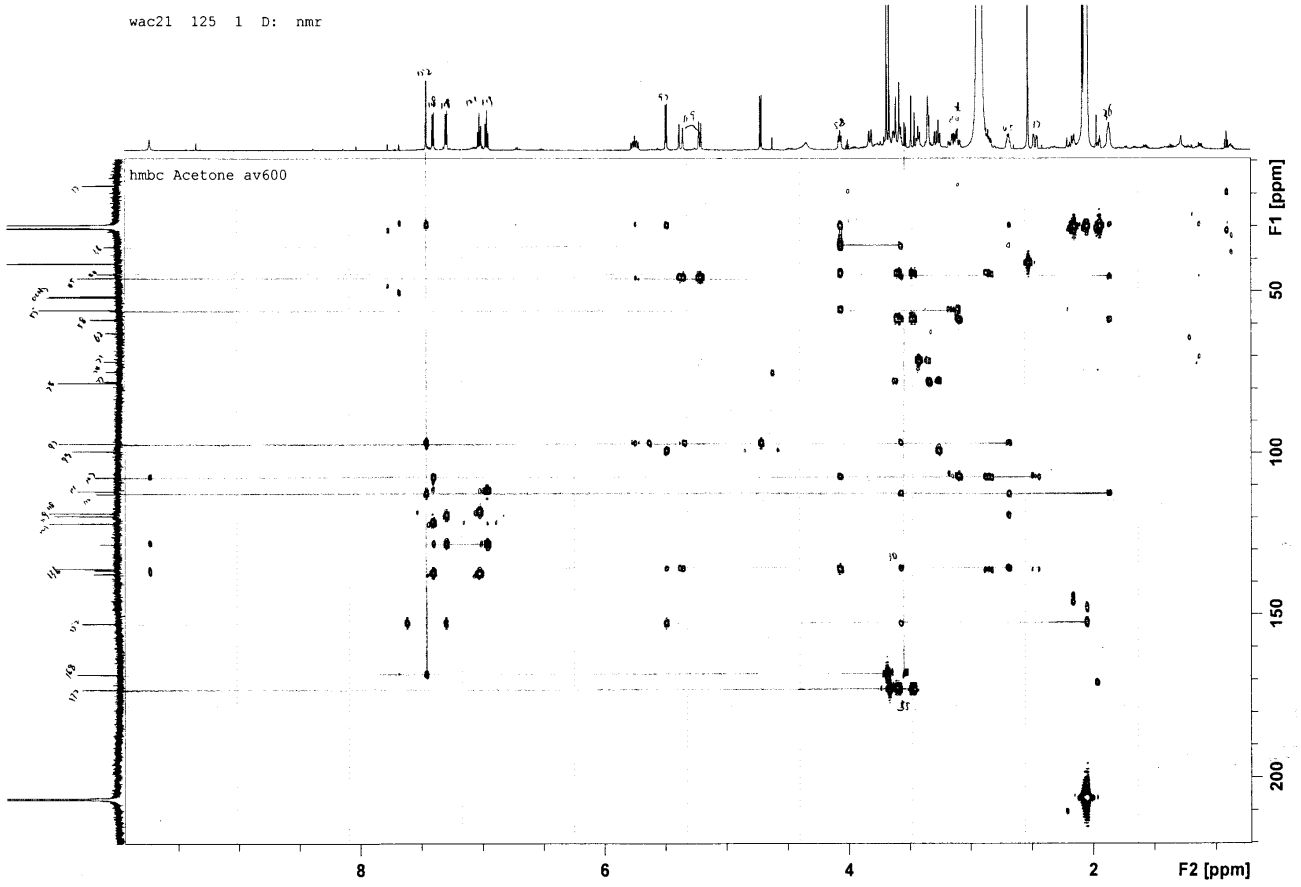

Figure 20. ROESY spectrum of alstrostine F (**4**)

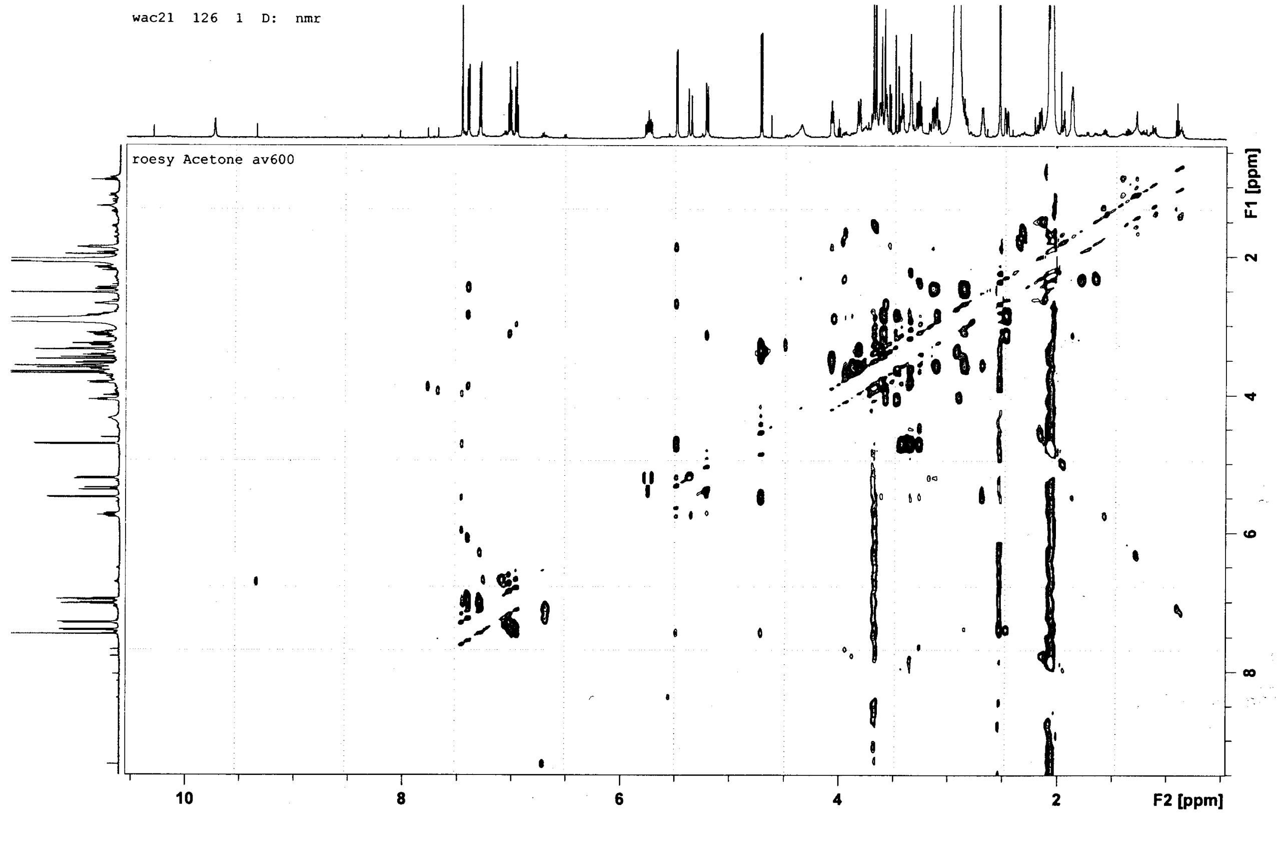

Supplement: Supplementary file 1 — Supplementary material, approximately 799 KB. [file 13659_2012_19_MOESM1_ESM.pdf]
